# Huaier n-butanol extract suppresses proliferation and metastasis of gastric cancer via c-Myc-Bmi1 axis

**DOI:** 10.1038/s41598-018-36940-w

**Published:** 2019-01-24

**Authors:** Yiping Wang, Hang Lv, Zhiyuan Xu, Jiancheng Sun, Yixiu Ni, Zhe Chen, Xiangdong Cheng

**Affiliations:** 1 Key Laboratory of Integrated Traditional Chinese and Western Medicine for Diagnosis and Treatment of Digestive System Tumor, the First Affiliated Hospital of Zhejiang Chinese Medical University, 54 Youdian Road, Hangzhou, 310006 China; 2 Department of Gastrointestinal Surgery, the First Affiliated Hospital of Zhejiang Chinese Medical University, Hangzhou, Zhejiang China

## Abstract

Gastric cancer (GC) ranks as the third leading cause of cancer-related mortality worldwide, and approximately 42% of all cases diagnosed each year worldwide are diagnosed in China. A large number of clinical applications have revealed that *Trametes robiniophila* Μurr. (Huaier) exhibits an anti-tumour effect. However, loss of the bioactive components of Huaier during the extraction procedure with water is unavoidable, and the underlying mechanism of the anti-cancer effect of Huaier remains poorly understood. In this study, we investigated the anti-cancer effect of Huaier n-butanol extract, which contained 51.4% total flavonoids, on HGC27, MGC803, and AGS human GC cell lines *in vitro*. At a low concentration, Huaier n-butanol extract inhibited the growth of these GC cell types, induced cell cycle arrest and reduced cell metastasis. Moreover, Huaier n-butanol extract suppressed the c-Myc-Bmi1 signalling pathway, and overexpression of Bmi1 reversed the effects of Huaier n-butanol extract on GC cells. Thus, our findings indicate that Huaier n-butanol extract suppresses the proliferation and metastasis of GC cells via a c-Myc-Bmi1-mediated approach, providing a new perspective for our understanding of the anti-tumour effects of Huaier. These results suggest that Huaier n-butanol extract could be an attractive therapeutic adjuvant for the treatment of human GC.

## Introduction

Cancer is currently one of the most important public health problems in the world. Despite improvements in diagnosis, surgical techniques, health care, and adjuvant therapy in recent years, which are all aimed at decreasing cancer mortality, carcinomas greatly attribute to human death. Gastric cancer (GC) ranks as the third leading cause of cancer-related mortality worldwide^1^, and approximately 42% of all cases diagnosed each year are diagnosed in China^2,3^. However, most GC patients are diagnosed with advanced disease and are no longer indicated for curative treatment^3,4^. Moreover, recurrence and metastasis are recognised as the most important obstacles to the therapeutic effects and poor patient survival of GC, and most deaths result from metastatic gastric carcinomas that are resistant to conventional therapies. Therefore, studies on the mechanism of GC metastasis and clinical trials of new drugs that could provide more effective therapy have attracted increasing attention.

Traditional Chinese medicine (TCM) has a long history of three millennia in Asia^5^. Due to its synergistic therapeutic effects and non-significant toxicity in cancer therapy, an increasing number of TCM components have been explored as alternative or complementary anti-cancer agents. *Trametes robiniophila* Μurr. (Huaier), a traditional Chinese herbal medicine, has been used in TCM for approximately 1,600 years. Currently, *Trametes robiniophila* bodies are extracted with water or alkali at different temperatures^6^. The effective ingredients of aqueous Huaier extract have been analysed by high-performance liquid chromatography. Proteoglycans were identified as the major components, consisting of 41.53% polysaccharides, 12.93% amino acids, and 8.72% water^7,8^. Aqueous Huaier extract exhibits anti-tumour effects in several cancers^9^. Increasing evidence suggests that Huaier exerts its anti-neoplastic activities by inhibiting proliferation, inducing apoptosis, suppressing angiogenesis, and inhibiting metastasis of cancer cells^9–13^. However, the underlying mechanism of the anti-cancer effect of Huaier remains poorly understood.

We previously demonstrated that aqueous Huaier extract inhibited cell proliferation, reversed drug resistance, and suppressed metastasis in GC^14,15^. However, the use of a high concentration reduces the effectiveness and universality of aqueous Huaier extract. Moreover, the efficiency of water extraction depends on the polarity of the targeted compounds. The bioactive compounds isolated by water extraction are mainly anthocyanins, tannins, saponins, and terpenoids^16,17^. Many active components are not water-soluble and are thus difficult to extract. In addition, temperature influences the bioactivity and composition of water extracts, including the loss of volatile components and the destruction of heat-sensitive ingredients. Therefore, loss of the bioactive components of Huaier during extraction with water is unavoidable. In light of these issues, we improved the extraction method and performed the extraction using different solvents, yielding five organic phases: petroleum ether, ethylacetate, n-butanol, an ethanol phase, and a water phase. *In vitro* cell experiments demonstrated that Huaier extract inhibits the proliferation of human GC MKN-45 cells. The most effective site is the locus of n-butanol, which inhibited GC MKN45 cell proliferation at a lower concentration than aqueous Huaier extract^18,19^. Further studies demonstrated that total flavonoids were the major component, with 51.4% in Huaier n-butanol extract. Flavonoids are a group of more than 4000 polyphenolic compounds, including flavones, flavanols, isoflavones, flavonols, flavanones, and flavanonols, *et al*.^20^. Flavonoids play important roles in cancer chemoprevention and chemotherapy. Many mechanisms of action have been identified, including carcinogen inactivation, anti-proliferation, cell cycle arrest, induction of apoptosis and differentiation, inhibition of angiogenesis, anti-oxidation, and reversal of multidrug resistance, or a combination of these mechanisms^21–23^. For example, flavonoids from licorice extract may be useful chemopreventive agents for peptic ulcers or GC in *Helicobacter pylori*-infected individuals^24^. Sophoranone, extracted from the TCM *Shan Dou Gen*, inhibited cell growth and induced apoptosis in various cancer cell lines, including human stomach cancer MKN7 cells and human leukaemia U937 cells^25^. Generally, more than 50% of a certain component in the extract of TCM can be considered the main component of the extract. Due to the anti-cancer effects of flavonoids, we hypothesise that flavonoids, in part, are responsible for the anti-cancer activity of Huaier n-butanol extract.

In this study, we investigated the anti-cancer effect of Huaier n-butanol extract on HGC27, MGC803, and AGS human GC cell lines *in vitro*. At a low concentration, Huaier n-butanol extract inhibited the growth of these GC cell types by inducing cell cycle arrest and reducing cell metastasis. Moreover, the expression of c-Myc and Bmi1 was largely suppressed when cells were exposed to Huaier n-butanol extract. Overexpression of Bmi1 reversed the effects of Huaier n-butanol extract on GC cells, indicating that Huaier n-butanol extract suppresses proliferation and metastasis in GC cells via a c-Myc-Bmi1-mediated signalling pathway. In addition, analysis of the expression of Bmi1 in 74 GC patient samples indicated that high expression of Bmi1 in GC tissues predicted lower disease-free survival (DFS). Taken together, these results suggest that Huaier n-butanol extract could be an attractive therapeutic adjuvant for the treatment of human GC.

## Results

### Huaier suppressed GC cell proliferation by inducing cell cycle arrest

**Figure 1:**
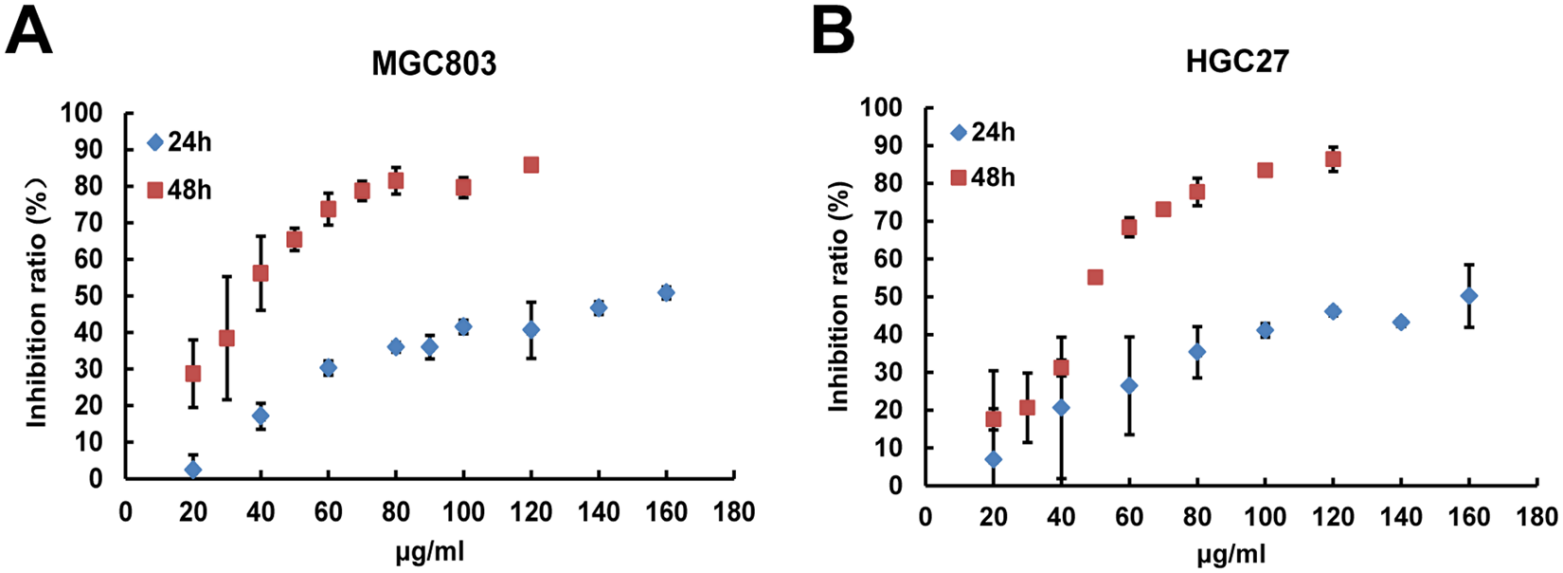
Huaier n-butanol extract inhibited gastric cancer (GC) cell proliferation *in vitro*. The growth inhibitory effect of Huaier n-butanol extract was measured using the CCK-8 assay. MGC803 (**A**) and HGC27 (**B**) cells were treated with varying concentrations of Huaier n-butanol extract (from 0 to 160 μg/ml for 24 and 48 h). The experiments were performed in triplicate, and the data are presented as the mean ± standard deviation (SD) of three separate experiments.

To examine the anti-cancer effects of Huaier n-butanol extract on GC, MGC803 and HGC27 cells were treated with varying concentrations of Huaier n-butanol extract (from 0 to 160 μg/ml for 24 and 48 h) and cell proliferation was assessed by the CCK-8 assay. As shown in Fig. 1A,B, inhibition of the two cell lines increased with increasing concentrations and exposure time, indicating that Huaier n-butanol extract suppresses GC cell proliferation in a time- and dose-dependent manner.

**Figure 2:**
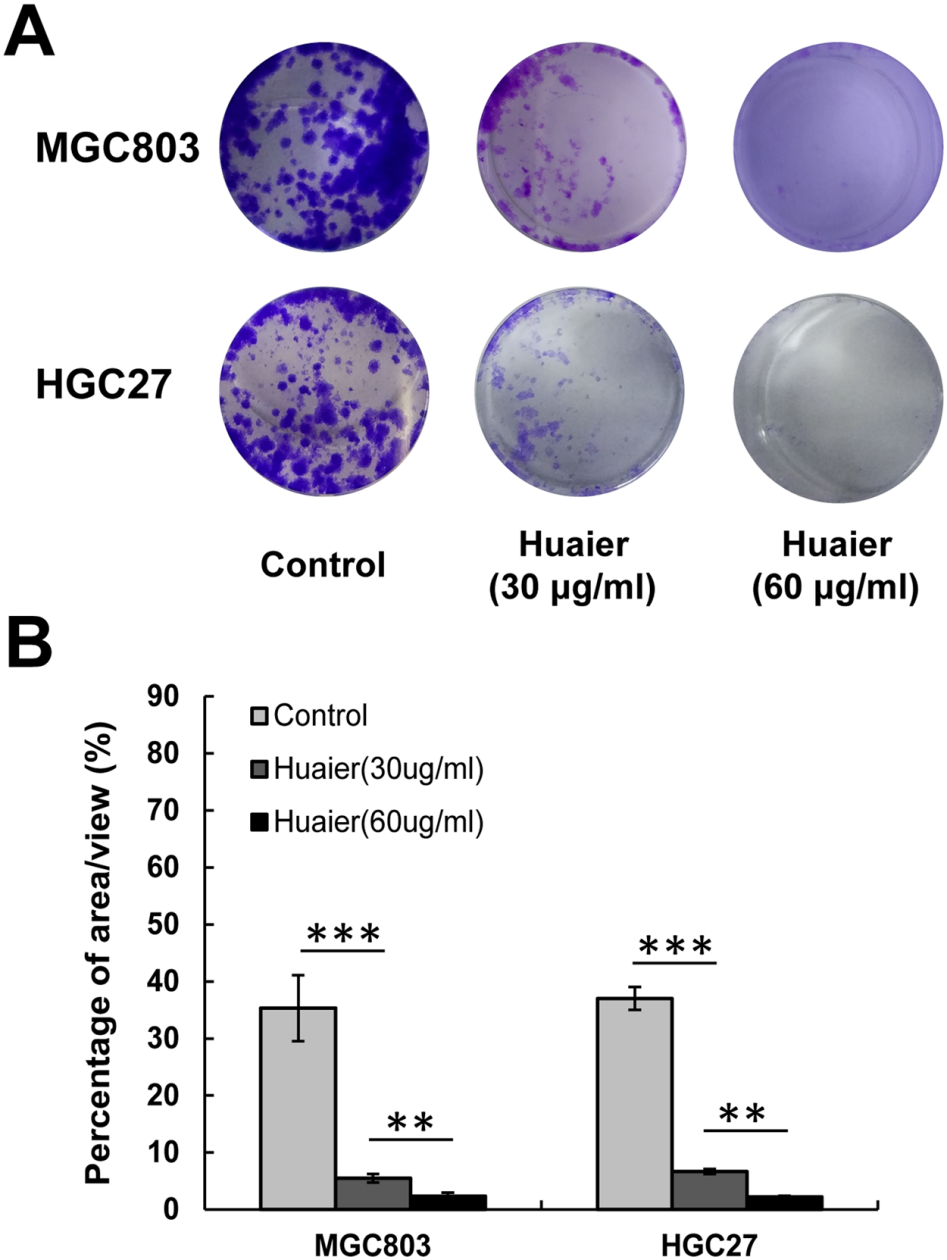
Huaier n-butanol extract inhibited the colony formation of GC cells. (**A**) Representative images of cell colonies after treatment with various concentrations of Huaier n-butanol extract. The colony formation of MGC803 and HGC27 cells was significantly reduced with increasing concentrations of Huaier n-butanol extract treatment. (**B**) The colony formation rate after treatment with Huaier n-butanol extract was calculated as the percentage of area covered by colonies per view (n = 3, bar graphs are plotted as the mean ± SD, **p < 0.01, ***p < 0.001, Student’s *t*-test).

Next, we performed colony formation assays to further determine the inhibitory effects of Huaier n-butanol extract on GC cell proliferation. Exposure to Huaier n-butanol extract reduced the numbers and size of the colonies formed by the two tumour cell lines in a dose-dependent manner (Fig. 2A). The area per view covered by colonies formed by cells treated with Huaier n-butanol extract or control (dimethyl sulphoxide [DMSO]) was compared, as shown in Fig. 2B.

**Figure 3:**
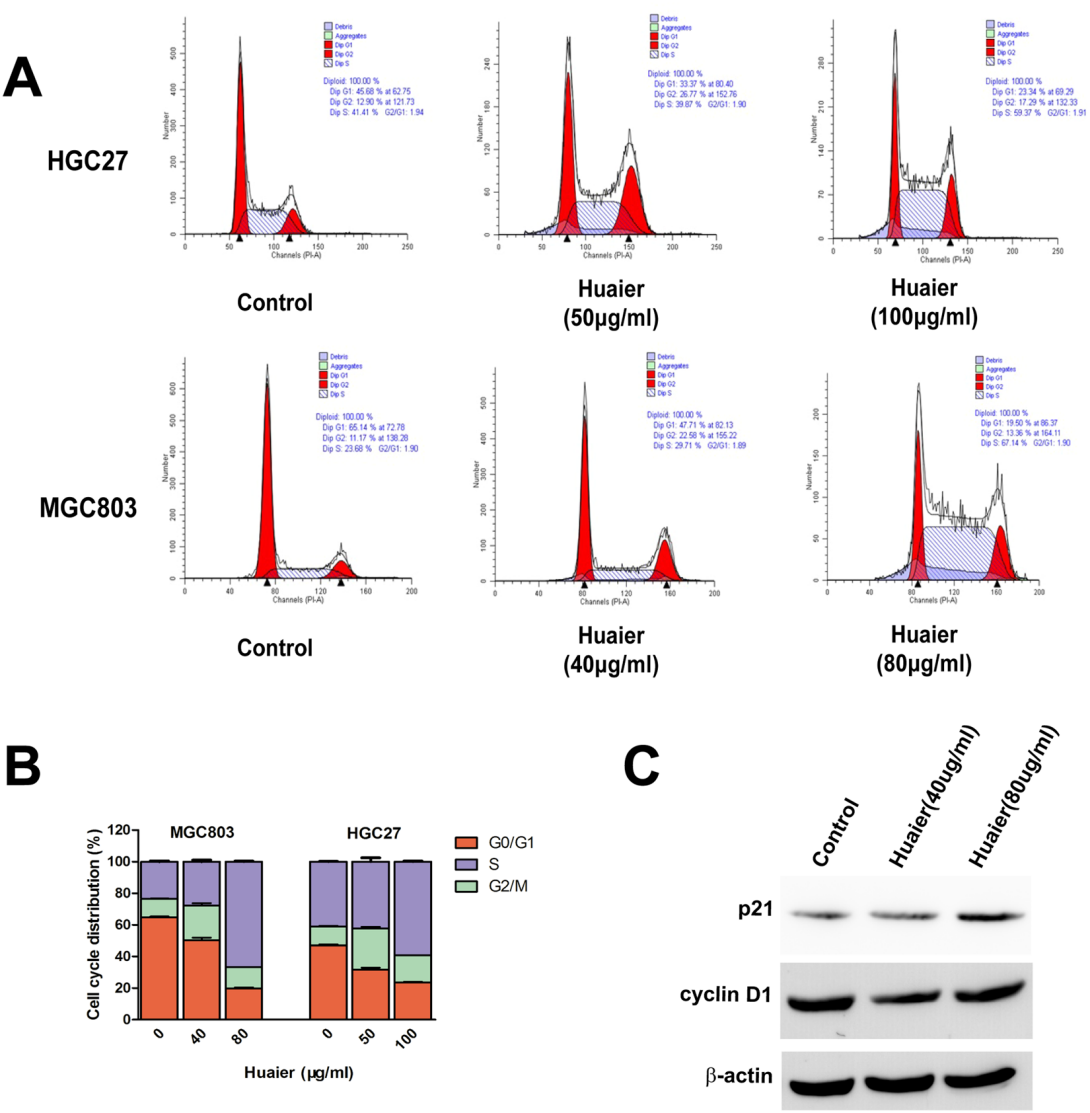
Huaier n-butanol extract induced cell cycle arrest in GC cells. (**A**) MGC803 and HGC27 cells were treated with gradient concentrations of Huaier n-butanol extract. Fluorescence-activated cell sorting analysis showed that cells accumulated in S and G2/M phases. (**B**) Quantitative analysis of cell cycle distribution. Cell cycle distribution of MGC803 and HGC27 cells was analysed. Data are presented as the mean ± SD of three independent experiments. (**C**) Effects of Huaier n-butanol extract on the expression of p21 and cyclin D1 in GC cells. HGC27 GC cells were treated with 0, 40, and 80 μg/ml of Huaier n-butanol extract for 48 h, and cell lysates were subjected to western blot analysis with p21 and cyclin D1 antibodies.

To investigate the possible mechanism of Huaier n-butanol extract on the inhibitory effects of GC cell proliferation, we investigated the effect of Huaier n-butanol extract on cell cycle regulation by flow cytometry in MGC803 and HGC27 GC lines. As shown in Fig. 3A,B, the cell population decreased at G0/G1 but increased at S and G2/M phases in both cell lines treated with Huaier n-butanol extract compared with control cells.

Next, major proteins associated with cell cycle progression were analysed by western blotting. The results in Fig. 3C show that p21, an essential negative regulator of the cell cycle involved in the G1-S cell cycle transition, increased in a dose-dependent manner in both cell lines after Huaier n-butanol extract treatment. Cyclin D1, which plays a critical role in the S/G2 transition, decreased after Huaier n-butanol extract treatment. Thus, these data are consistent with the increase at S and G2/M phases in the flow cytometric analysis. Taken together, we conclude that Huaier n-butanol extract exerts its anti-cancer activities by blocking cell cycle progression.

### Huaier n-butanol extract inhibited GC cell invasion and migration

**Figure 4:**
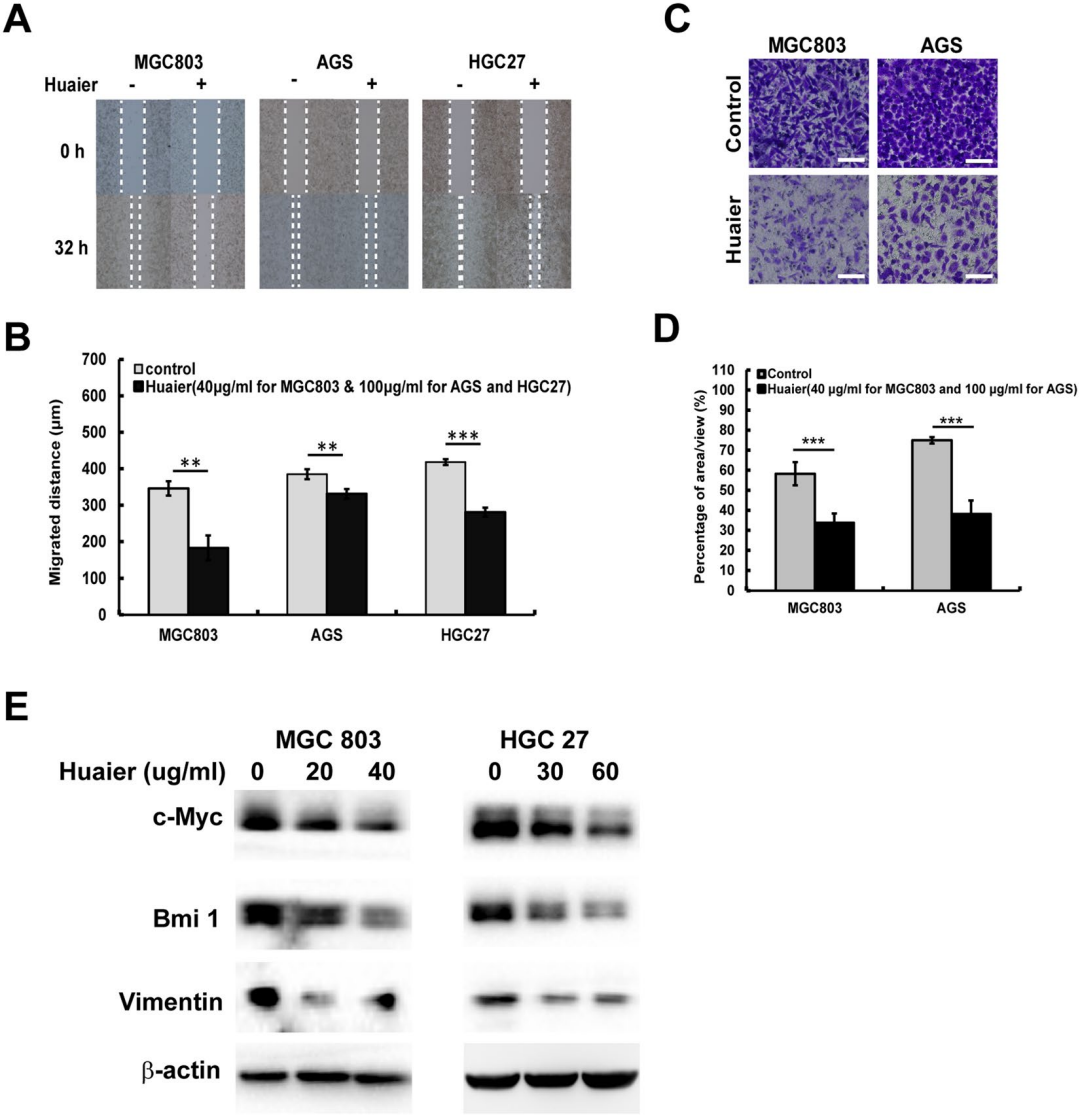
Huaier n-butanol extract suppressed GC cell migration and invasion by regulating the c-Myc-Bmi1 signalling pathway. (**A**) Detection of migration by the wound-healing assay. (**B**) Quantitative analysis of migration speed using the migration index (n = 3, bar graphs are plotted as the mean ± SD, **p < 0.01, ***p < 0.001, Student’s *t*-test). (**C**) Detection of cell invasion using high-throughput screening multi-well insert 24-well two-chamber plates. White bars represent 100 μm. (**D**) Quantitative analysis of invaded cells (n = 3, bar graphs are plotted as the mean ± SD, ***p < 0.001, Student’s *t*-test). (**E**) Immunoblot analysis of the expression of vimentin, c-Myc, and Bmi1 using the corresponding antibodies.

To assess the effect of Huaier n-butanol extract on cellular motility, we conducted wound-healing assays to determine cell migration speed. The migration speed of MGC803, HGC27, and AGS cells was significantly decreased following exposure to Huaier n-butanol extract relative to the control groups (Fig. 4A,B). We also performed transwell assays to measure the invasion of MGC803 and AGS cells. Huaier n-butanol extract significantly reduced the number of GC cells that invaded through the Matrigel in the bottom chamber, as shown in Fig. 4C,D.

Since the epithelial-mesenchymal transition (EMT) plays an important role in promoting cell migration and invasion, we further determined the expression level of the EMT-related protein vimentin to explore the potential mechanism by which Huaier n-butanol extract reduces metastasis in GC cells. Following exposure to Huaier n-butanol extract for 48 h, the expression level of vimentin was reduced in a dose-dependent manner in both cell lines compared with control cells (Fig. 4E). Based on these results, Huaier n-butanol extract appears to halt GC cell invasion and migration by suppressing the expression of vimentin.

### Huaier n-butanol extract induced downregulation of the c-Myc-Bmi1 signalling pathway in GC cells

Due to the abnormal expression of many genes involved in the process of gastric carcinogenesis and development, Huaier has multiple targets in the anti-tumour process. In the current study, Huaier n-butanol extract interfered with the c-Myc-Bmi1 signalling pathway. As shown in Fig. 4E, the expression of c-Myc was suppressed in MGC803 and HGC27 cells treated with Huaier n-butanol extract. Moreover, the expression of Bmi1, which is regulated by c-Myc^26^, was also decreased by Huaier n-butanol extract treatment, suggesting that the c-Myc-Bmi1 signalling pathway plays an important role in the anti-cancer effect of Huaier n-butanol extract.

### Overexpression of Bmi1 reversed the effects of Huaier n-butanol extract on GC cells

**Figure 5:**
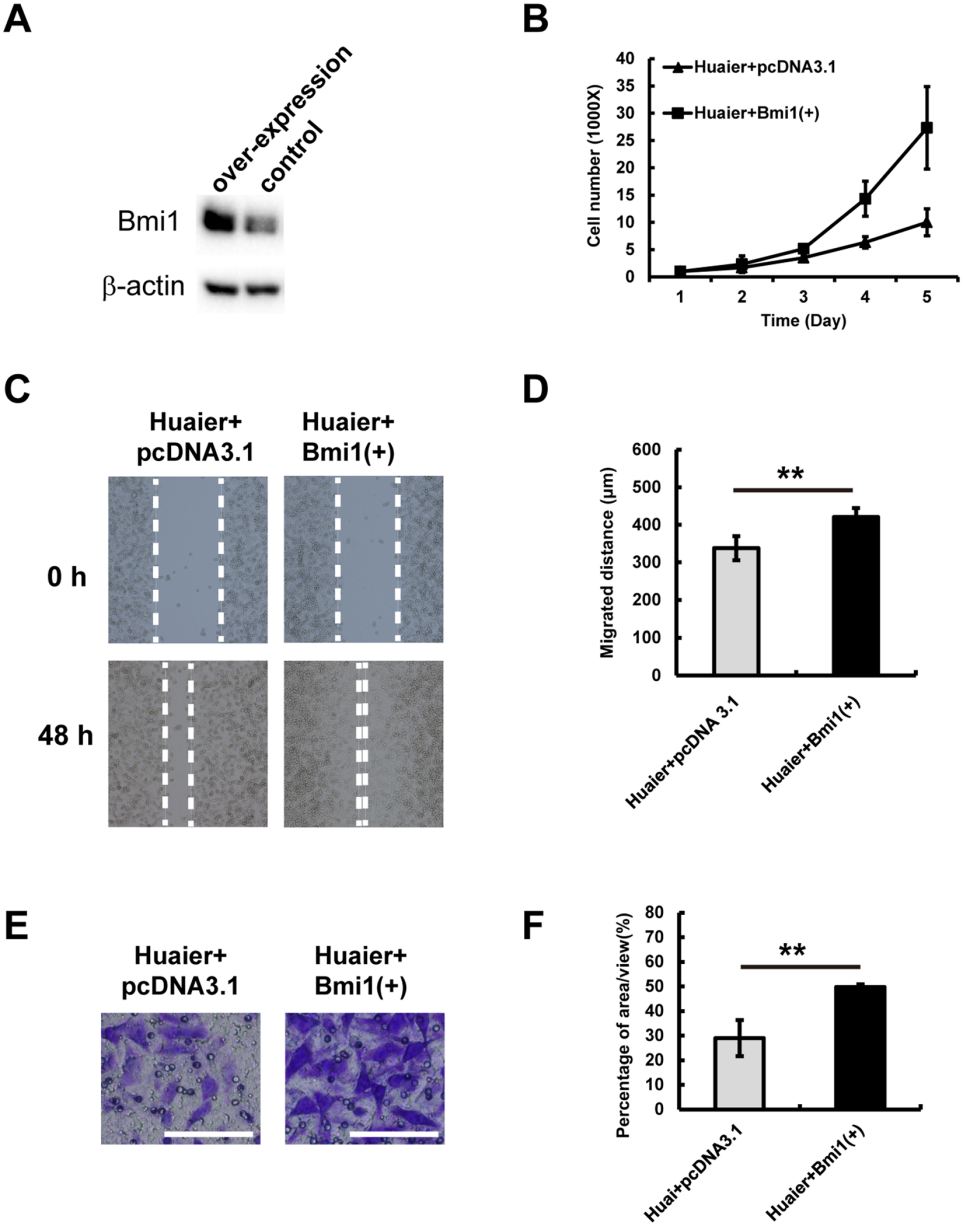
Overexpression of Bmi1 reversed the effects of Huaier n-butanol extract on GC cells. (**A**) MGC803 GC cells were transfected with a Bmi1 plasmid for 48 h. Cell lysates were subjected to immunoblot analysis to examine the expression of Bmi1. (**B**) Proliferation of MGC803 cells overexpressing Bmi1 or the negative control. (**C**) MGC803 cells transfected with the negative control or the Bmi1 plasmid were subjected to wound healing assays and observed at 0 and 48 h. (**D**) Quantitative analysis of the migration speed using the migration index (n = 3, bar graphs are plotted as the mean ± SD, **p < 0.01, Student’s *t*-test). (**E**) MGC803 cells transfected with the negative control or the Bmi1 plasmid were subjected to transwell assays. White bars represent 100 μm. (**F**) Quantitative analysis of invaded cells (n = 3, bar graphs are plotted as the mean ± SD, **p < 0.01, Student’s t-test).

To explore whether the decreased expression of Bmi1 induced by Huaier n-butanol extract was responsible for the suppression of proliferation and metastasis of GC cells following exposure to Huaier n-butanol extract, we investigated the effects of Bmi1 expression on the proliferation and metastasis of MGC803 cells. As shown in Fig. 5A, Bmi1 expression was upregulated in MGC803 cells following transfection with a Bmi1 plasmid. Increased expression of Bmi1 significantly accelerated the proliferation of MGC803 cells (Fig. 5B). Moreover, MGC803 cells transfected with the Bmi1 plasmid exerted enhanced invasive and migratory capacities (Fig. 5C–F). Collectively, we propose that the downregulation of Bmi1, at least in part, contributes to the inhibition of proliferation and metastasis of GC cells following treatment with Huaier n-butanol extract.

### Bmi1 is highly expressed in GC tissues and correlates with cancer differentiation

**Figure 6:**
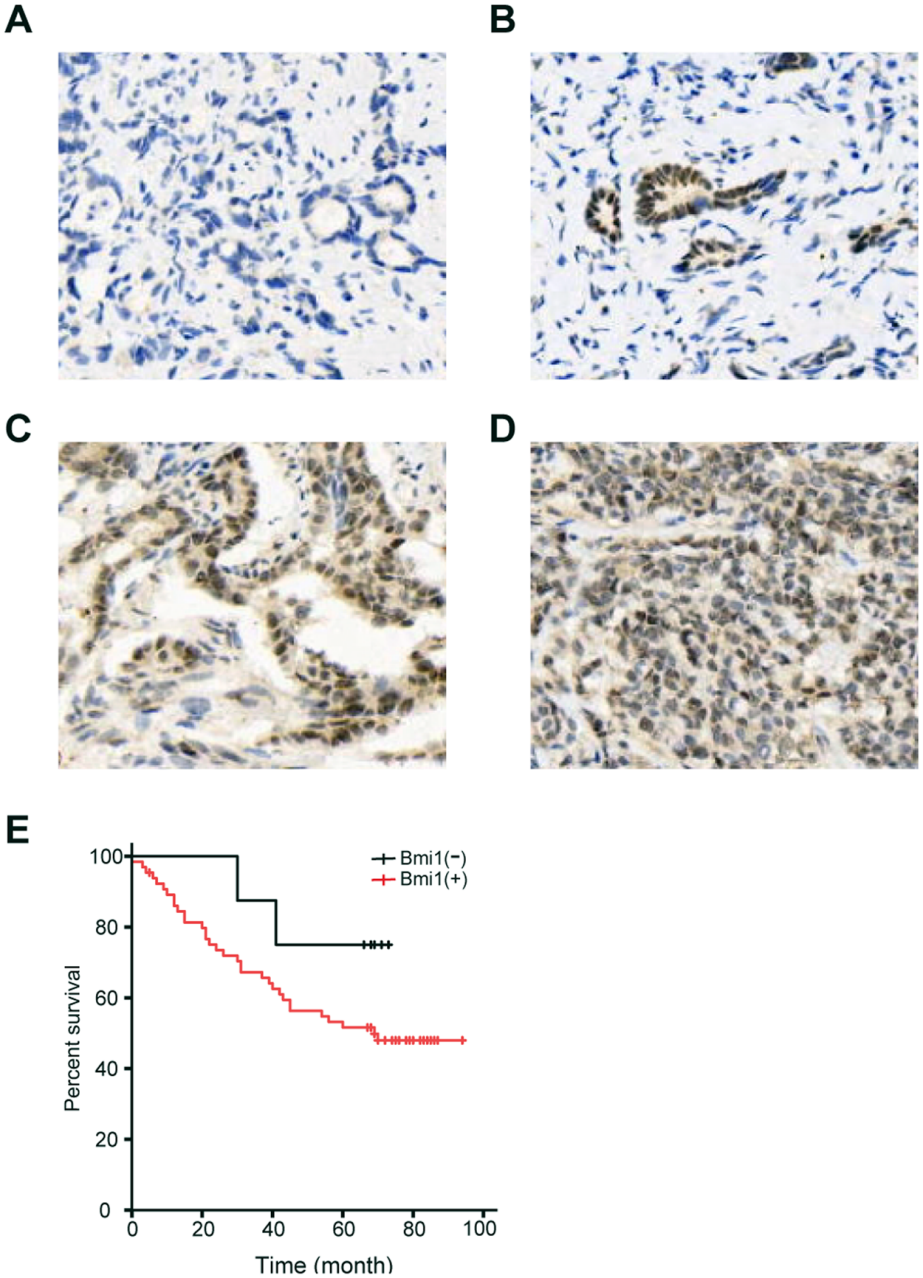
Immunohistochemical staining for Bmi1 in GC tissues. (**A**) No staining in GC tissues. (**B**) Weak staining in GC tissues (1+). (**C**) Moderate staining in GC tissues (2+). (**D**) Strong staining in GC tissues (3+). (**E**) Disease-free survival curves of GC patients according to Bmi1 expression level.

To explore the therapeutic value of Huaier n-butanol extract in GC patients, we determined whether Bmi1 expression was increased in gastric tumours. We examined tissues from 74 GC patients (Fig. 6A–D). Among all cases, 8 (10.8%) were Bmi1-negative (0 to 1+) and 66 (89.2%) were Bmi1-positive (2+ to 3+); of these, 26 cases (35.1%) were positive (++) and 40 (54.1%) were strongly positive (+++). The positive expression level of Bmi1 in GC tissues was significantly correlated with the grade of differentiation (P = 0.015). There was no significant correlation between Bmi1 expression and age, sex, tumour size, tumour location, lymph node metastasis, venous invasion, advanced TNM stage, or Lauren’s classification (Table 1). The 3- and 5-year cumulative DFS rates were 66.7% and 51.5%, respectively, for patients with high Bmi1 expression, and 87.5% and 75%, respectively, for those with low Bmi1 expression (Fig. 6E).

**Table 1:** The relationship between Bmi1 expression and clinicopathological features of in 74 GC patients.

Clinicopathologic factors	Number of each group	Bmi1 expression		*P* value
		Low	High	
All case	74	8	66	
Age (years)				0.591
<60	34	4	30	
≥60	40	4	36	
Gender				0.893
Male	56	5	51	
Female	18	3	15	
Tumor size (cm)				0.529
<5	34	7	27	
≥5	40	1	39	
Location of tumor				0.961
Cardia	7	1	6	
Body	9	0	9	
Antrum	25	3	22	
More than two parts	33	4	29	
Depth of tumor invasion				0.391
T1	4	2	2	
T2	11	2	9	
T3	13	1	12	
T4	46	3	43	
Lymph node metastasis				0.429
N0	2	0	2	
N1	17	1	16	
N2	19	3	16	
N3	36	4	32	
Venous invasion				0.654
Absent	32	5	27	
Present	42	3	39	
Stage				0.164
I,II	16	4	12	
III,IV	59	4	55	
Lauren’s classification				0.921
Diffuse	60	4	56	
intestinal	14	4	10	
Grade of differentiation				0.015
Well/moderate	35	4	31	
Poor/not	39	4	35	

## Discussion

TCM has become a widely discussed topic due to the potential anti-tumour properties of its components and is an important source for the development of new anti-tumour drugs due to low toxicity, low cost, and multiple targets^27^. Huaier has been used in TCM for approximately 1,600 years. Clinical applications and reports have confirmed that aqueous Huaier extract inhibits proliferation, induces apoptosis, inhibits migration, and reverses drug resistance in several types of solid tumours^28–30^. Huaier suppresses proliferation and induces apoptosis in human pulmonary cancer cells by upregulating miR-26b-5p^31^. Aqueous Huaier extract significantly restrains the proliferative and migratory potential of hepatocellular carcinoma cells by decreasing yes-associated protein 1^32^. However, the exact mechanism remains unclear, and the active ingredients of Huaier have not been completely delineated, limiting the potential for drug development. Hence, analysing and obtaining the active ingredients of Huaier, as well as exploring the anti-tumour mechanisms of Huaier, have become highly necessary. We extracted Huaier using different solvents and obtained Huaier n-butanol extract, which contains 51.4% total flavonoids^19^. Further experiments demonstrated that Huaier n-butanol extract inhibited GC MKN45 cell proliferation at a lower concentration than aqueous Huaier extract^18^.

In the present study, Huaier n-butanol extract inhibited the growth of human MGC803 and HGC27 GC cells in a time- and dose-dependent manner, and the IC_50_ value after 48-h treatment was 35.3 µg/ml in MGC803 cells and 46.3 µg/ml in HGC27 cells. Compared to aqueous Huaier extract purchased from Gaitianli Medicine Co. Ltd. (Jiangsu, China), the survival ratio of MGC803 cells, at a concentration of 0.2 mg/ml, was 67.4% after treatment for 48 h^33^, indicating that Huaier n-butanol extract exerts stronger effects on GC cell proliferation than aqueous Huaier extract. Next, we performed a series of experiments to determine the anti-cancer properties of Huaier n-butanol extract on GC cell lines. *In vitro* assays demonstrated that Huaier n-butanol extract inhibited the proliferation of GC cells and induced cell cycle arrest at S and G2/M phases. Moreover, the present study investigated the expression of proteins involved in cell cycle progression, including cyclin D1 and p21, by western blot analysis. Huaier n-butanol extract-induced cell cycle arrest was, at least in part, caused by the deactivation of cyclin D1 and the upregulation of p21.

In addition, Huaier n-butanol extract significantly inhibited MGC803, HGC27, and AGS cell migration and invasion, consistent with the detection of protein expression levels by western blotting. The decreased expression of vimentin, an EMT-related hallmark, demonstrated that treatment with Huaier n-butanol extract suppressed migration and invasion in GC cells.

The underlying mechanism of the anti-tumour effect of Huaier remains less well characterised. AKT/GSK3β/β-catenin, ERα, and JNK/p38 signalling play important roles in the inhibition of proliferation and metastasis of human cancer cells by Huaier^28,30,34^. The protein encoded by the proto-oncogene c-Myc is one of the most frequently affected genes in human cancers^35–37^ and is regarded as a “master regulator” that plays important roles in human cell growth and metabolism^38^. c-Myc is expressed at a low level in normal cells and becomes deregulated or significantly elevated in most human cancers. Activation of c-Myc is crucial for sustained tumour cell proliferation and survival^39–42^, while suppression of c-Myc expression induces tumour regression in different tumour types^43^ and promotes rapid tumour deterioration by triggering apoptosis or senescence^44^. In this study, the expression of c-Myc was suppressed when GC cells were exposed to Huaier n-butanol extract, suggesting its therapeutic potential. Moreover, the c-Myc oncogene has long been believed to execute its neoplastic functions by acting as a classic transcription factor, deregulating the expression of a large number of target genes. B-lymphoma mouse Moloney leukaemia virus insertion region 1 (Bmi1), a member of the polycomb group, is a direct target of c-Myc that regulates the transcription of Bmi1 by binding to the E-box element within its promoter^26^. Bmi1 is a transcription repressor that plays essential roles in the regulation of stem cell self-renewal, embryogenesis, cell proliferation, and senescence^45–47^. Increasing evidence has demonstrated that Bmi1 is highly expressed and involved in the pathogenesis of various aggressive cancers, including breast, neuroblast, colon, and esophagus^48–50^. Elevated expression of Bmi1 in colon cancer patients is important for the self-renewal of colon cancer stem cells and promotes the invasion and migration of colon cancer cells^51,52^. Bmi1 increases the level of p-Akt and enhances tumour cell proliferation, migration, and anti-apoptotic abilities in the human breast carcinoma cell line MCF-7^53^. In the present study, Huaier n-butanol extract suppressed the expression of c-Myc and Bmi1 in a dose-dependent manner. Immunohistochemical analysis of 74 GC patient samples indicated that positive expression of Bmi1 in GC tissues was significantly correlated with the grade of differentiation (P = 0.015). A higher expression level of Bmi1 was correlated with lower DFS.

In conclusion, we showed that Huaier n-butanol extract inhibited cell proliferation, colony formation, migration, and invasion in GC by downregulating c-Myc and Bmi1. The c-Myc-Bmi1 axis may be applied to advance our knowledge regarding GC pathogenesis, and Huaier n-butanol extract may have implications for the development of treatment strategies for GC.

## Methods

### Patient samples

The study was approved by the First Affiliated Hospital of Zhejiang Chinese Medical University. 74 cases of GC patients were recruited in this study. All participants have given written informed consent, and all studies involving human samples were performed in accordance with guideline regulation of the standards of the Ethics Committee at Zhejiang Cancer Hospital, specifically pertaining to IRB Number: 2016-123.

### Cell lines and reagents

Human gastric cancer (GC) cell lines, including HGC27, MGC803 and AGS, were obtained from the Cell Bank of the Chinese Academy of Science (Shanghai, China). HGC27, MGC803 and AGS cells were maintained in RPMI-1640 (Gibco®, Hangzhou MultiSciences Biotech Co., Ltd., Hangzhou, China) supplemented with 10% fetal bovine serum (Hyclone) at 37 °C in a humidified atmosphere of 5% CO_2_. The antibodies included p21, cyclin D1, vimentin, Bmi1, c-Myc, and β-actin were purchased from Abcam (Cambridge, UK).

### Preparation of Huaier

Huaier n-butanol extraction was performed as described previously^18,19^. Huaier was purchased from Anhui (Bozhou Medical Materials Co. Ltd., Anhui, China). The fruiting bodies of Huaier were powdered and reflux-extracted with heated 90% ethanol twice (2 h each time). The extract solution was filtered and combined, evaporated, and concentrated to a thick paste. After vacuum drying, 90% ethanol extract powder was obtained. Subsequently, 90% ethanol extract powder was extracted with petroleum ether, ethyl acetate, n-butanol, 90% ethanol, and distilled water sequentially. Five different powders of Huaier extract were obtained by concentrating and recovering the solvent on a rotary evaporator, freezing, and vacuum drying. Huaier n-butanol extract was dissolved in DMSO and sterilised with a 0.22-μm filter to obtain a 100 mg/ml stock solution and stored at −20 °C. Fresh dilutions in medium were made for each experiment.

### *In vitro* cytotoxicity

The *in vitro* cytotoxicity of Huaier was measured by Cell Counting Kit-8(CCK-8) (Dojindo, Japan), as described in the manufacturer’s protocol. Briefly, 5 × 10^3^ cells per well were plated in 96-well plates and treated with Huaier or DMSO (diluent) at various concentrations for 24 or 48 h. Then, the medium with Huaier or DMSO was replaced with 200 μL of fresh medium along, 10 μL CCK-8 solution was added into each well and incubated at 37 °C for 2 h. Absorbance was measured at 450 nm using a spectrophotometer (Bio-Rad, USA).

### Colony forming assay

1 × 10^3^ cells were seeded into each well on a 6 cm Petri dish at a single cell density and treated with different concentrations of Huaier n-butanol extract for 24 h. The cells were cultured for another 14 days and the medium was refreshed every three days. Thereafter, the cells were washed with phosphate buffer saline (PBS) and were fxed with methanol, dried and stained with 0.1% crystal violet. Images of clones were obtained using an Olympus digital camera (Olympus, Tokyo, Japan). The percentage of area covered by cells per view was calculated with ImageJ.

### Cell cycle analysis

3 × 10^5^ cells were seeded into each well on a 6 cm Petri dish the day before. The cells were collected after incubated with Huaier n-butanol extract for 48 h. The cells were fixed with 75% cold ethanol at −20 °C overnight. Then, the cells were stained with 200 of μL RNase A (1 mg/mL) and 500 μL of propidium iodide (PI, 100 μg/mL) (Liankebio, Zhejiang, China) for 30 min at room temperature in the dark, and they were analyzed using a FACScan flow cytometer. The data were analysed using ModFitLT sofware, version 2.0 (Becton Dickinson, Franklin Lakes, NJ, USA).

### Invasion assay

Cell invasion assay was performed using a transwell plate (BD Biosciences, San Jose, CA) and followed the manufacturer’s protocol. 5 × 10^4^ cells were placed in the upper chamber, the cells treated with Huaier n-butanol extract were allowed to invade at 37 °C for 72 hours toward a lower reservoir. The stained cells were counted under an inverted microscope. Each experiment was performed in triplicate.

### Wound healing assay

Wound healing assays was performed by using the ibidi Culture–Insert 2 Well. 70 ul of Cells (5 × 10^5^/ml) were implanted onto each well. After cells grew to 90% confluence (24 hours) gently remove the Culture–Insert 2 Well by using sterile tweezers. The remaining cells were incubated at RPMI-1640 supplemented with 2% FBS and Huaier n-butanol extract. At the indicated times, migrating cells were photographed, the migration distance at indicated time compared with time 0 was analyzed using Image-Pro Plus version 6.2 software.

### SDS polyacrylamide gel electrophoresis and Western blot analysis

Proteins were extracted from the whole cells using RIPA buffer (150 mM sodium chloride, 0.5% sodium deoxycholate, 1% Triton X100, 0.1% sodium dodecyl sulfate SDS, and 50 mM Tris-HCl pH 8) with protease inhibitor cocktail and PMSF. Equal amounts of cell lysates were separated by SDS-PAGE and transferred to a nitrocellulose membrane (Life Sciences). The membranes were probed with primary antibodies overnight at 4 °C, followed by incubation with corresponding secondary antibodies conjugated with horseradish peroxidase. The immunoreactivity was visualized by enhanced chemiluminescence (WesternBright Quantum, Menlo Park, CA).

### Immunohistochemistry

GC tissues were fixed in 10% formalin, then paraffin-embedded and sliced into 4 μm sections. Immunohistochemistry was performed according to the manufacturer’s protocol. Briefly, paraffin-embedded slides were deparaffinized, rehydrated and washed in 1% PBS-Tween, then treated with 3% hydrogen peroxide and blocked with 10% goat serum for 1 h at 37 °C. Slides were incubated with primary antibodies in PBS containing 1% BSA (1:200) overnight at 4 °C. Biotinylated secondary anti-rabbit (or mouse) antibodies were added and incubated at room temperature for 1 h. Streptavidin-HRP was added, and after 40 min, the sections were stained with DAB substrate and counter stained with hematoxylin.

The slides were evaluated independently by two pathologists in a blind fashion. The cells with brown-colored staining were considered as positive. The intensity of Bmi1 expression was stratified into four categories that were scored as follows: (1) 0, negative, no staining; (2) 1+ had appreciable staining in 1 to 25% of cells; (3) 2+ had 25 to 50%; and (4) 3+ had >50% of the specimen stained. A score of 2+ and 3+ was considered to be a positive IHC result.

### Statistical analysis

Results were presented as means ± SD. Each set of experiment was repeated at least three times. Analysis was performed using one-way ANOVA and Student’s t-test. Difference was considered to be significant if *p* < 0.05.

## Supplementary information

supplementary information
