# Supplementary material for: Huaier n-butanol extract suppresses proliferation and metastasis of gastric cancer via c-Myc-Bmi1 axis
Source: Sci Rep. 2019 Jan 24;9:447. doi: 10.1038/s41598-018-36940-w (PMC6346047; doi:10.1038/s41598-018-36940-w)

Supplementary Information to the manuscript

Huaier n-butanol Extract Suppresses Proliferation and Metastasis of Gastric Cancer via c-Myc-Bmi1 Axis

Yiping Wang[1], Hang Lv[1], Zhiyuan Xu[1,2], Jiancheng Sun[2], Yixiu Ni[2], Zhe Chen[1], Xiangdong Cheng[1,2]*

[1] Key Laboratory of Integrated Traditional Chinese and Western Medicine for Diagnosis and Treatment of Digestive System Tumor, the First Affiliated Hospital of Zhejiang Chinese Medical University,54 Youdian Road, Hangzhou, 310006, China

[2] Department of Gastrointestinal Surgery, the First Affiliated Hospital of Zhejiang Chinese Medical University, Hangzhou, Zhejiang, China

To whom correspondence should be addressed: Xiangdong Cheng, Key Laboratory of Integrated Traditional Chinese and Western Medicine for Diagnosis and Treatment of Digestive System Tumor, the First Affiliated Hospital of Zhejiang Chinese Medical University, 54 Youdian Road, Hangzhou, 310006, China. Tel.: Tel: +86-571-87070965; Fax: +86-571-87070965; E-mail: chengxd@zcmu.edu.cn

This file contains supplementary figure legends and two supplementary figures.

Supplementary figure legends

Supplementary figure 1 Full-length Western blots appeared in the main manuscript

The full-length Western blots of Figure 3C and Figure 5A are included.

The rectangle boxes indicate the areas of the Western blots shown in the main figures. MW kDa-molecular weights in kiloDaltons of the marker proteins. AB indicates the antibody used for the blot.

Supplementary figure 2 Full-length Western blots appeared in the main manuscript

The full-length Western blots of Figure 4E are included. The rectangle boxes indicate the areas of the Western blots shown in the main figures. MW kDa: molecular weights in kiloDaltons of the marker proteins. AB indicates the antibody used for the blot.

Supplementary figure 1

Figure 3C

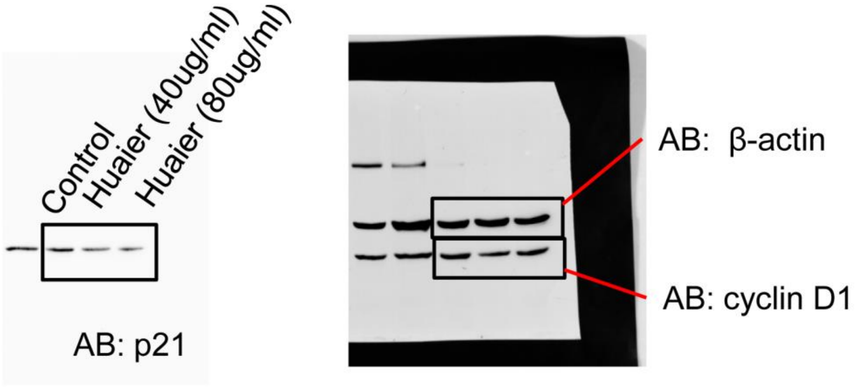

Figure 5A

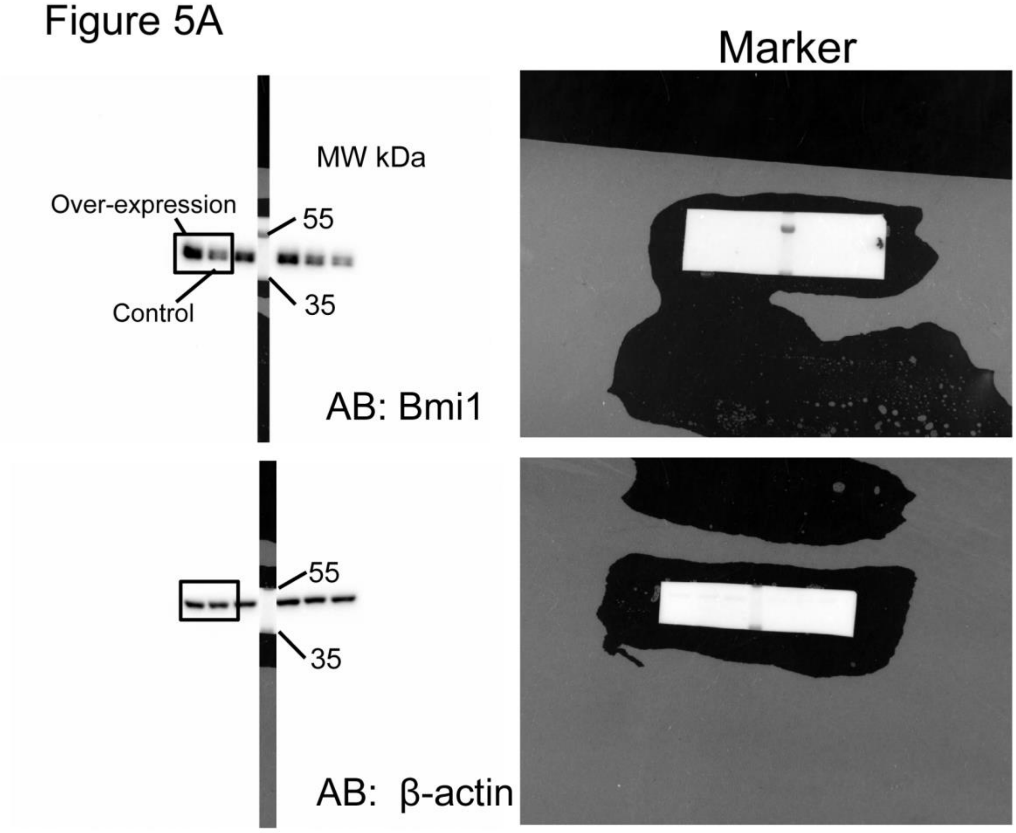

Supplementary figure 2

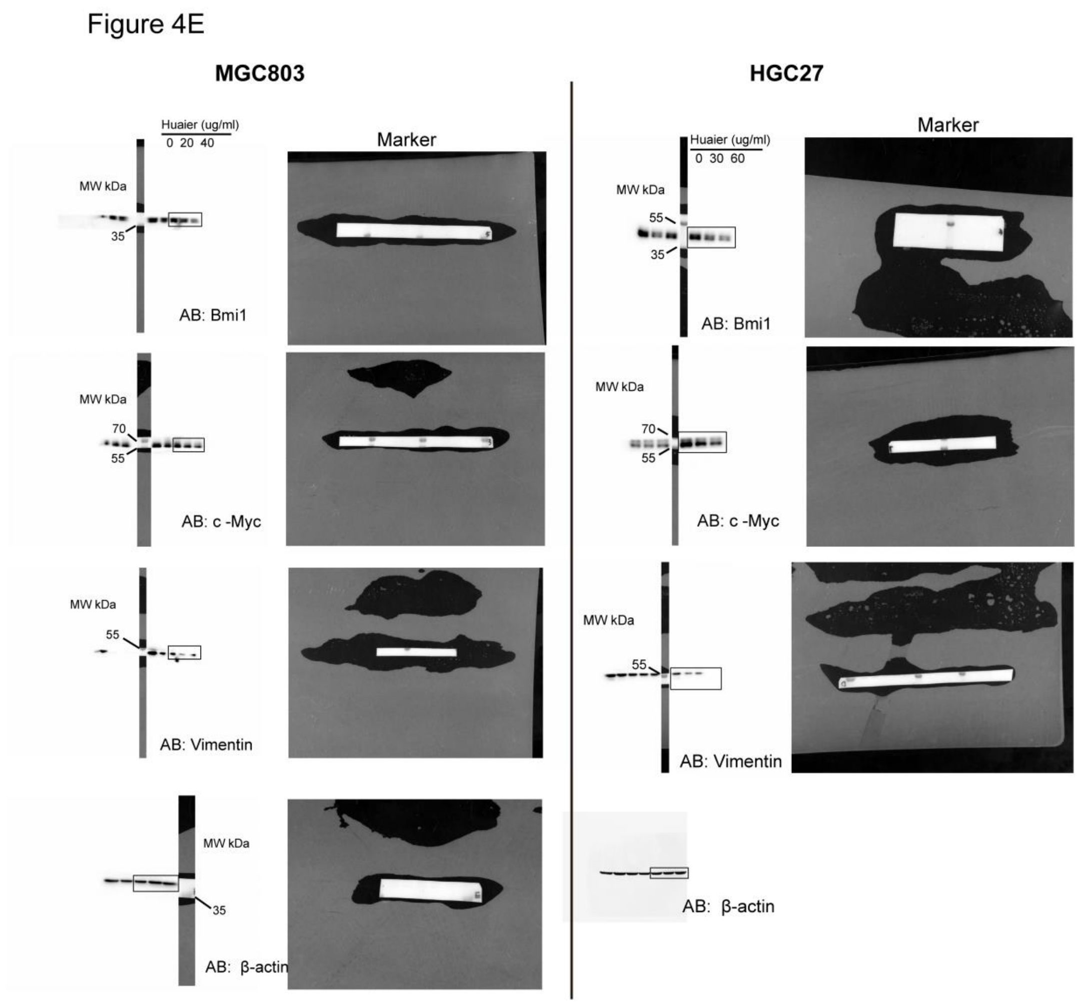

Supplement: Supplementary file 1 — supplementary information [file 41598_2018_36940_MOESM1_ESM.pdf]
